# Analysis of the *Caulobacter crescentus* Zur regulon reveals novel insights in zinc acquisition by TonB-dependent outer membrane proteins

**DOI:** 10.1186/1471-2164-15-734

**Published:** 2014-08-28

**Authors:** Ricardo Ruiz Mazzon, Vânia Santos Braz, José Freire da Silva Neto, Marilis do Valle Marques

**Affiliations:** Departamento de Microbiologia, Instituto de Ciências Biomédicas, Universidade de São Paulo, Av. Prof. Lineu Prestes 1374, 05508-900 São Paulo, Brazil; Departamento de Biologia Celular e Molecular e Bioagentes Patogênicos, Faculdade de Medicina de Ribeirão Preto, Universidade de São Paulo, Av. Bandeirantes, 3900, Ribeirão Preto, 14049-900 São Paulo, Brazil

**Keywords:** *Caulobacter crescentus*, Zur regulon, Zinc homeostasis, TonB-dependent receptor

## Abstract

**Background:**

Intracellular zinc concentration needs to be maintained within strict limits due to its toxicity at high levels, and this is achieved by a finely regulated balance between uptake and efflux. Many bacteria use the Zinc Uptake Regulator Zur to orchestrate zinc homeostasis, but little is known regarding the transport of this metal across the bacterial outer membrane.

**Results:**

In this work we determined the *Caulobacter crescentus* Zur regulon by global transcriptional and *in silico* analyses. Among the genes directly repressed by Zur in response to zinc availability are those encoding a putative high affinity ABC uptake system (*znuGHI*), three TonB-dependent receptors (*znuK*, *znuL* and *znuM*) and one new putative transporter of a family not yet characterized (*zrpW*). Zur is also directly involved in the activation of a RND and a P-type ATPase efflux systems, as revealed by β-galactosidase and site-directed mutagenesis assays. Several genes belonging to the Fur regulon were also downregulated in the *zur* mutant, suggesting a putative cross-talk between Zur and Fur regulatory networks. Interestingly, a phenotypic analysis of the *znuK* and *znuL* mutants has shown that these genes are essential for growth under zinc starvation, suggesting that *C. crescentus* uses these TonB-dependent outer membrane transporters as key zinc scavenging systems.

**Conclusions:**

The characterization of the *C. crescentus* Zur regulon showed that this regulator coordinates not only uptake, but also the extrusion of zinc. The uptake of zinc by *C. crescentus* in conditions of scarcity of this metal is highly dependent on TonB-dependent receptors, and the extrusion is mediated by an RND and P-type ATPase transport systems. The absence of Zur causes a disturbance in the dynamic equilibrium of zinc intracellular concentration, which in turn can interfere with other regulatory networks as seen for the Fur regulon.

**Electronic supplementary material:**

The online version of this article (doi:10.1186/1471-2164-15-734) contains supplementary material, which is available to authorized users.

## Background

Zinc, as it occurs with other transition metals, entails a paradox to living cells. It is a scarce micronutrient in the environment, essential for a substantial number of biological processes [1]. On the other hand, despite not being a redox-active metal, in excess zinc may cause significant toxicity due to its high competitiveness relative to other divalent cations from the Irving–Williams series [2]. Thus, it is extremely necessary to maintain intracellular zinc levels under tight regulation. Bacterial zinc homeostasis is accomplished by a coordinated control of uptake, storage and export, which is mainly a result of controlled expression of import and export systems, each one governed by its own regulatory system [3].

A large number of bacteria regulate zinc uptake through Zur (Zinc Uptake Regulator), a regulatory protein belonging to the Fur (Ferric Uptake Regulator) family. This metalloprotein contains one structural zinc ion and a second one in its regulatory site per monomer, and acts as a repressor of genes coding for zinc uptake systems under zinc sufficiency [4, 5]. More recently, it was showed that when loaded with metal in the regulatory site the protein may act as a direct repressor or activator binding to the promoter region of regulated genes [5]. The induction of extrusion systems is also a recurring response when zinc is in excess inside the cell. Two families of regulators, ArsR and MerR, were first described as the most important factors responsible for induction of these extrusion systems [4]. The P-type ATPase ZntA from *Escherichia coli* is regulated by the MerR-like transcriptional regulator ZntR [6]. CzrB from *Staphylococcus aureus* is a Cation Difusion Facilitator (CDF) involved in zinc extrusion that is regulated by CzrA, an ArsR-like transcriptional repressor [7]. More recently, other studies have reported that extrusion systems are also regulated in response to zinc in a Zur-dependent manner, as described for *Xanthomonas campestris* and *Corynebacterium glutamicum* [8–10].

Zinc enters the periplasmic space either via outer membrane non-specific porins or via TonB dependent receptors such as *Neisseria meningitidis* ZnuD and CbpA [11, 12], and in some cases the free metal may be sequestered by metalloproteins present in this compartment [1]. Once in the periplasm, zinc can cross the cell membrane through high affinity ABC transporters such as ZnuABC in *E. coli* [13] and YciABC in *Bacillus subtilis* [14], broad-spectrum cation transporters like MntH in *E. coli* [15], metal ion transporters of ZIP superfamily such as ZupT in *E. coli* [16] or as a neutral metal phosphate through constitutively expressed inorganic phosphate uptake system (Pit) [17]. At least three types of transport systems have been described for the extrusion of zinc ions when in excess: P-type ATPases such as *E. coli* ZntA [18], RND (Resistance-Nodulation-Division) type exporters such as *Alcaligenes eutrophus* CzcABC [19] and CDF such as *S. aureus* ZntA [20].

Although zinc homeostasis has been extensively studied in a great diversity of bacterial groups, there are few studies in bacteria belonging to the group of α-proteobacteria. The α-proteobacteria group clusters organisms with widely different lifestyles. Within this group there are representatives of human and animal pathogens such as *Rickettsia*, *Brucella* and *Ehrlichia*, specimens of agronomic importance like *Agrobacterium tumefaciens* and many aquatic and marine species [21]. Considering the great importance of biological processes involving zinc carried out by bacteria of this group, such as photosynthesis in *Rhodobacter sphaeroides* [22] and virulence of *B. abortus* [23], understanding zinc homeostasis in α-proteobacteria is highly relevant.

*Caulobacter crescentus* is a free-living and oligotrophic α-proteobacterium widely spread through aquatic environments and very well adapted to utilize the scarce nutrients present in these environments. This bacterium has been extensively studied due to its finely regulated asymmetric cell division, which results in the generation of a stalked cell and a motile cell [24]. Genome sequence analyses of this bacterium have identified only two genes encoding transcriptional regulators belonging to the Fur family and 65 genes encoding TonB-dependent receptors [25]. Previous works from our group have studied the Fur regulator of *C. crescentus*, demonstrating that its regulon contains many transport systems involved in iron acquisition, including several TonB-dependent receptors [26, 27]. Additionally, our group has characterized the LysR-type regulator CztR that activates the cell membrane transporter CztA, putatively involved in zinc acquisition [28].

In this work, we have investigated the contribution of the second regulator of the Fur family (here called Zur) for zinc homeostasis in *C. crescentus* via global transcriptomics analysis. The Zur regulon includes genes directly repressed by Zur encoding zinc uptake systems like high-affinity ABC transporters and TonB-dependent receptors, and a Zur-activated operon encoding an RND efflux system and a P-Type ATPase. Our results also indicate that in the absence of Zur there is an *in vivo* interference in the expression of some genes of the Fur regulon. Furthermore, we present experimental evidence that two zinc and Zur-regulated TonB-dependent receptors are essential for growth under zinc starvation, showing their importance for zinc uptake across the bacterial outer membrane.

## Results

### Construction of a *C. crescentus zur*mutant strain

There are two genes encoding transcriptional regulators belonging to the Fur family annotated in the *C. crescentus* genome: the well-characterized *fur* gene, involved in iron homeostasis [26, 27] and CC0357, which could encode the zinc uptake regulator Zur. Alignment of *E. coli*, *Yersinia pestis*, *X. campestris* and *Pseudomonas aeruginosa* Zur proteins with the protein encoded by CC0357 shows sequence conservation in Zur-specific residues which are not shared with other members from the Fur family, like Fur and Nur [see Additional file 1: Figure S1]. This protein shares 46%, 43%, 45% and 47% identity with *E. coli*, *Y. pestis*, *X. campestris* and *P. aeruginosa* Zur proteins respectively. Based on these alignments and the experimental findings described throughout the text, we here call this protein Zur. To determine the role of this regulator in zinc homeostasis and *C. crescentus* physiology, a *zur* deleted strain (MM69), was obtained by allelic exchange. This strain showed growth rates in rich and minimal medium similar to the wild-type strain, and a 10% increase in intracellular zinc content (*zur*: 0.433 ± 0.08 ppm/*wt*: 0.392 ± 0.09 ppm). Oxidative stress survival in MM69 strain was evaluated and no increased susceptibility to oxidative agents as hydrogen peroxide, pyrogalol, tert-butil hydroperoxide or paraquat was observed for the *zur* mutant in M2 medium (data not shown).

### Determination of the Zur regulon using *in silico*and global transcriptomics analyses

To identify the Zur regulon in response to zinc availability, total RNA from NA1000 (wild type) and MM69 was extracted 1 h after addition of 200 μM ZnCl_2_ to both cultures. The results showed that 28 genes had their expression altered in MM69, being 7 upregulated and 21 downregulated in the mutant (Table 1). Three genes included in Table 1 (CC0663, CC2720 and CC2722) were differentially expressed in the *zur* strain but did not fully attend the cutoff criteria; however, they were included because CC0663 presents a 100% conserved *in silico* predicted Zur-binding sequence and CC2720 and CC2722 belong to the Zur-regulated putative operon CC2720-26. Upregulated genes include those potentially involved in zinc uptake, such as those encoding an ABC-2 type transport system here called *znuGHI* (CC1518-CC1519-CC1520), three TonB-dependent receptors here called *znuK*, *znuL* and *znuM* (CC1517, CC0214 and CC0663) and a hypothetical protein with six transmembrane domains (CC0320), that could be a putative zinc transporter (here called *zrpW*, for Zinc Responsive Protein W). A zinc-requiring cobalamin-independent methionine synthase (CC0482) was also upregulated in the *zur* mutant.

**Table 1:** **Genes differentially expressed in the** ***zur*** **mutant**

Gene number	Gene name	Predicted Zur binding sequence	Predicted function^a^	Fold change (***zur***/wt)
**Upregulated**				
CC0214	*znuL*	TACGTTATTTCATAACAGT	TonB-dependent receptor	5.02
CC0320	*zrpW*	AATGTTACTTTATAACACG	Hypothetical protein (putative transporter domain)	8.15
CC0482			5-methyltetrahydropteroyltriglutamate/homocysteine S-methyltransferase (EC:2.1.1.14)	2.33
CC0663^**b**^	*znuM*	CCTGTTACAGAATAACAGG	TonB-dependent receptor	7.21
CC1517	*znuK*	GATGTTATATCATAACAAT	TonB-dependent receptor	41.30
CC1518	*znuG*	ATTGTTATGATATAACATC	ABC-2 type transport system ATP-binding protein	29.39
CC1519	*znuH*		ABC-2 type transport system permease protein	38.09
CC1520	*znuI*		ABC-2 type transport system permease protein	11.61
**Downregulated**				
CC0027			PKHD-type hydroxylase (2OG-Fe(II) oxygenase superfamily)	0.31
CC0711	*feoA*		Ferrous iron transport protein A	0.45
CC1775			Hypothetical protein (DUF2946 domain)	0.37
CC1776			Transcriptional regulator (GntR family)	0.48
CC1777	*sodA*		Superoxide dismutase, Fe-Mn family	0.23
CC1778			TonB-dependent receptor	0.44
CC1780			ThiJ/PfpI protease family protein	0.50
CC1782			Hypothetical protein	0.47
CC2192			Hypothetical protein (DUF3297 and Maf-like domains)	0.44
CC2193			Hypothetical protein	0.48
CC2720^**b**^		GCCGTAATTAAGTAACAGA	Hypothetical protein (Predicted periplasmic or external membrane component protein with DUF2946 domain)	0.46
CC2721	*czrC*		RND system	0.29
CC2722^**b**^	*czrB*		RND system	0.59
CC2723			Hypothetical protein (Predicted periplasmic protein)	0.46
CC2724	*czrA*		RND system	0.40
CC2725			Hypothetical protein (Predicted cytoplasmic membrane component with DUF190 domain)	0.38
CC2726	*zntA*		P-type ATPase	0.31
CC2727			Hypothetical protein (glutaredoxin domain)	0.40
CC2927			Hypothetical protein (PepSY-associated TM helix)	0.41
CC2928			TonB-dependent receptor	0.46
CC3060			Hypothetical protein (Predicted periplasmic protein with DUF2271 domain)	0.50
CC3061			Hypothetical protein (DUF4198 domain)	0.41
CC3263	*bfd*		Hypothetical protein (Fer2_BFD domain)	0.28

^**a**^Protein function is described as annotated at the KEGG website.

^**b**^These genes had altered expression in the *zur* mutant but did not reach the cutoff criteria established for differentially expressed genes.

Among the downregulated genes in the *zur* mutant, there are two large gene clusters, one containing genes for a GntR regulator, a Fe-Mn superoxide dismutase (SodA), a TonB-dependent receptor and hypothetical proteins (CC1775-76-77-78-80-82) and another cluster encoding an RND efflux system (*czrCBA*), a putative P-type ATPase (*zntA*) and hypothetical proteins (CC2720-21-22-23-24-25-26). A 500-nt fragment upstream to *zntA* was cloned upstream to the *lacZ* reporter gene and that fusion showed no β-galactosidase activity, indicating the absence of a promoter; therefore we assumed that *zntA* is cotranscribed with the CC2720-25 putative operon (data not shown). All the other remaining genes (CC0027, CC0711, CC2192-93, CC2927-28, CC3060-61 and CC3263) were previously described as belonging to the Fur regulon and are organized in clusters that contain at least one gene predicted to be involved in iron acquisition [26, 27] (Table 1).

To identify Zur-binding sites in the *C. crescentus* genome we performed two *in silico* analyses. Initially, a search using the motif GTTA-5 N-TAAC, conserved in Zur-binding sites of many proteobacteria was carried out. Only four putative Zur-binding sites with a 100% conserved motif were retrieved, increasing to 53 when one-base pair change was allowed. Comparing this *in silico* analysis with the Zur-regulated genes identified in the DNA microarray analysis, we found that only five upregulated and one downregulated genes/operons have a predicted Zur-binding site located upstream to the annotated translation start site (Table 1). Separately, an *ab initio* search, using the MEME tool with the upstream region of all Zur-regulated genes, identified the same six putative Zur-binding sites. Taken together, these data indicate that Zur directly represses five transcriptional units (CC0214, CC0320-21, CC0663, CC1517 and CC1518-19-20) encoding putative zinc uptake systems, and directly activates one putative operon (CC2720-21-22-23-24-25-26) encoding zinc efflux systems (Table 1). Most of the remaining genes downregulated in the *zur* mutant are not preceded by a predicted Zur-binding site and belong to the Fur regulon, suggesting that they are indirectly activated by Zur (see Discussion below).

### Validation of the zinc and Zur-regulated genes

**Figure 1:**
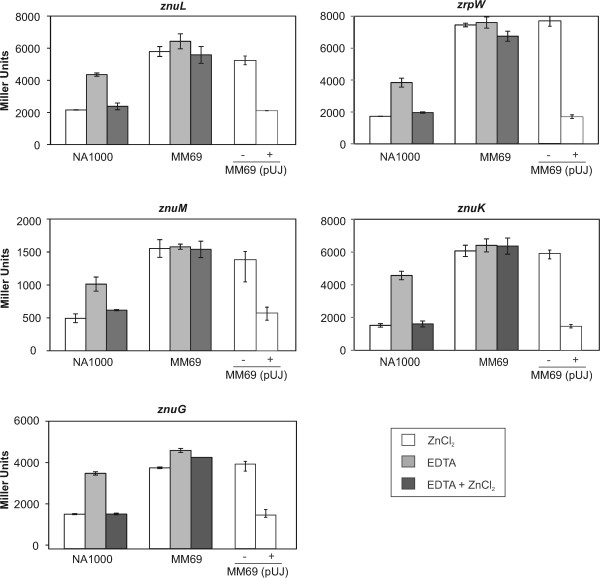
**Influence of Zur and zinc availability in the expression of Zur-repressed genes.** Transcriptional fusions of *znuK*, *znuL*, *znuM*, *zrpWX* and *znuGHI* promoter regions to the *lacZ* reporter gene were introduced into NA1000, MM69, MM69 (pUJ) and MM69 (pUJ-*zur*) strains, and grown under different regimens of zinc availability. Expression was determined by β-galactosidase activity assays. White bars indicate M2 medium supplied with 500 μM ZnCl_2_, light grey bars indicate M2 medium supplied with 500 μM EDTA and dark grey bars indicate M2 medium supplied with 500 μM ZnCl_2_ and 500 μM EDTA. In the complementation analysis, MM69 strain harboring plasmid pUJ142 without insert (−) or carrying the *zur* gene (+) was used. The results shown are the average of at least three experiments. Error bars indicate standard deviations.

To validate the Zur-regulated genes obtained in the microarray and *in silico* approaches, the regions upstream to the annotated start codons were amplified by PCR and cloned upstream to a *lacZ* reporter gene. Plasmids containing the transcriptional fusions were introduced into both the NA1000 and MM69 strains. β-galactosidase assays were performed in minimal media supplemented with 500 μM ZnCl_2_, 500 μM EDTA (simulating zinc depletion) or 500 μM of both (Figure 1). According to this assay, the expression driven by the promoters of *znuK*, *znuL*, *znuM*, *zrpW* and *znuGHI* increased in the zinc depletion condition triggered by EDTA and were derepressed in the *zur* mutant strain. The expression of these genes returned to the levels shown in the presence of zinc when both EDTA and ZnCl_2_ were added in NA1000 but not in the *zur* mutant, indicating that these genes are repressed by Zur in the presence of zinc, validating the microarray data. Complementation of the *zur* mutation with a copy of the *zur* gene *in trans* (plasmid pUJ-*zur*) restores the repression under excess zinc (Figure 1).

**Figure 2:**
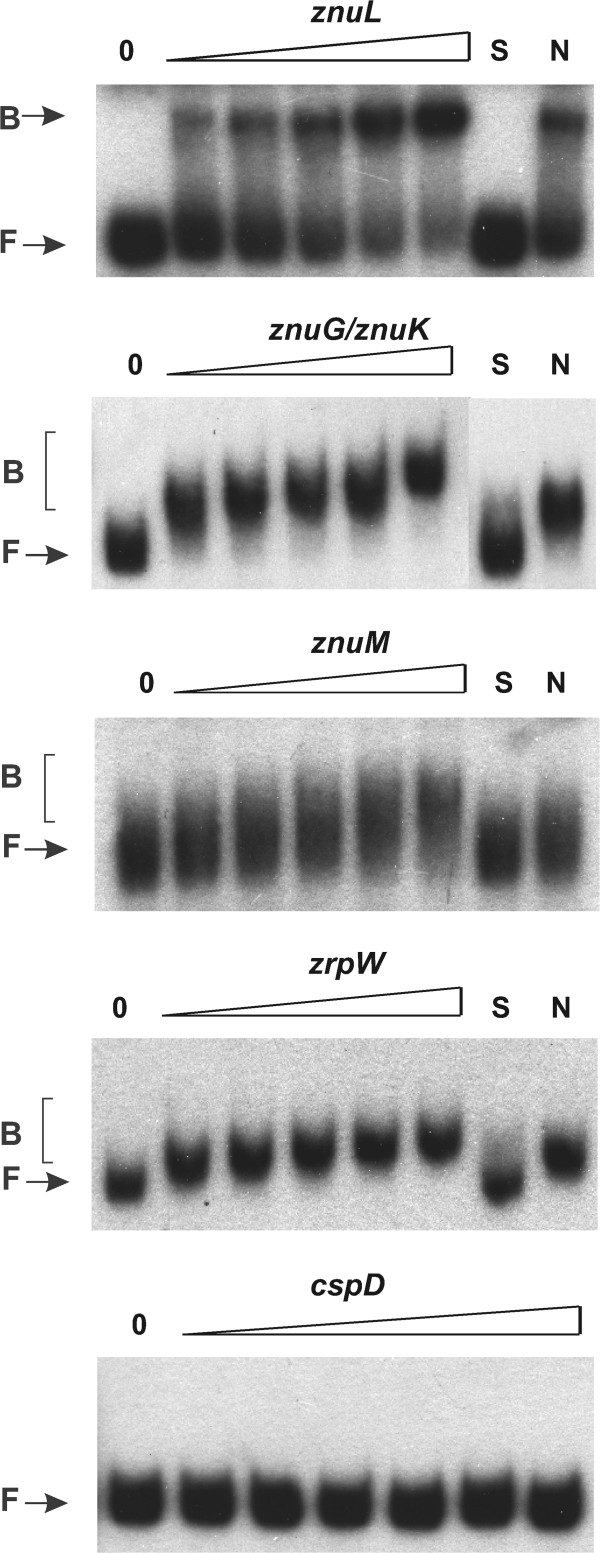
**Zur binding to the regulatory regions of Zur-repressed genes.** DNA fragments corresponding to the promoter regions of *znuL*, *znuM*, *zrpWX* and *znuK*/*znuGHI* and the coding region of *cspD* were labeled with ^32^P and incubated or not with increasing concentrations of purified His-FLAG-Zur (0, 25, 50, 100, 200, 500 and 1000 nM; this latter concentration was used only for *cspD*). Competition assays were carried out with 0.2 μM of purified His-FLAG-Zur in the presence of 30-fold of unlabeled probe (S) or in the presence of 30-fold of non-specific competitor (N). The non-specific competitor probe was the coding region of the stationary phase-induced gene *cspD*. Free probe and His-FLAG-Zur bound probe are indicated by (F) and (B), respectively.

The same fragments containing the gene promoters described above were used in EMSA with the *C. crescentus* purified His-FLAG-Zur protein (Figure 2). According to the results, Zur was able to bind *in vitro* to the promoter of *znuK*/*znuGHI*, *znuL, znuM* and *zrpW*, and the probes presented shifted mobility with addition of even the lowest concentration of Zur (25 nM). The specificity of Zur binding was demonstrated by absence of shift when the probe containing the coding region of the stationary phase-induced gene *cspD* was used, even at the highest Zur concentration (1 μM). Moreover, competition with excess unlabeled specific probe caused a complete loss of shift, which was not seen when excess of unlabeled nonspecific probe was added.

**Figure 3:**
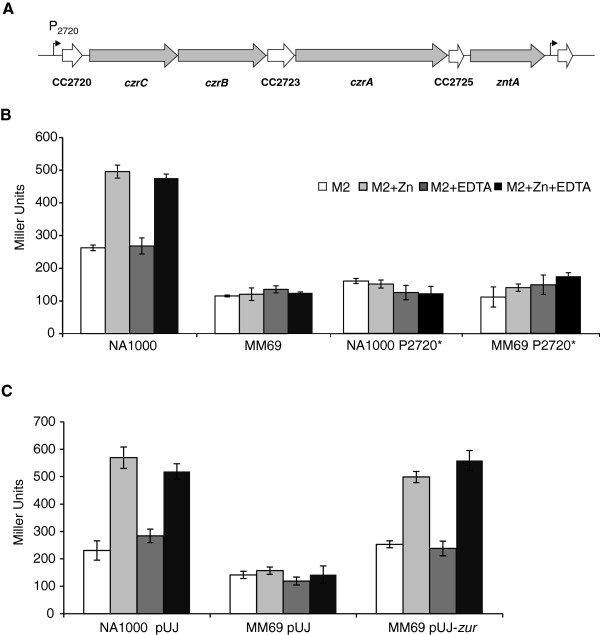
**Role of Zur in the expression of the CC2720-26 operon in response to zinc. (A)** Scheme of *C. crescentus* CC2720-26 *locus* depicting the putative *czrCBA* operon organization described in [29]. Gray arrows indicate genes encoding the RND and P-type ATPase transport systems. White arrows indicate hypothetical proteins. **(B)** β -galactosidase activity assay of CC2720-26 operon in response to zinc availability, in NA1000 (wild type) and MM69 (*zur* mutant) strains. White bars, M2 medium, light grey bars, M2 containing 500 μM ZnCl_2_, dark grey bars, M2 containing 500 μM EDTA and black bars, M2 containing both 500 μM ZnCl_2_ and 500 μM EDTA. Asterisks indicate that the putative Zur binding site into the promoter region of CC2720-26 was mutagenized. The results shown are the average of at least three experiments. Error bars indicate standard deviations. **(C)** β -galactosidase activity assay of CC2720-26 operon in response to zinc availability in NA1000 and MM69 strains harboring plasmid pUJ142 containing or not the *zur* gene.

The transcriptional *lacZ* fusion containing the promoter region of the *czrCBA* operon (CC2721, CC2722 and CC2724), encoding an RND efflux system, three hypothetic proteins (CC2720, CC2723 and CC2725) and the P-type ATPase ZntA (CC2726) (Figure 3A) was previously available [29]. The expression of *lacZ* driven by this promoter was low in cultures grown in M2 medium or M2 containing 500 μM EDTA and increased in the presence of 500 μM ZnCl_2_ or 500 μM of both (Figure 3B). Moreover, expression of this construct is very low in the *zur* mutant, indicating that this operon is activated by Zur in presence of zinc, validating the microarray data.

**Figure 4:**
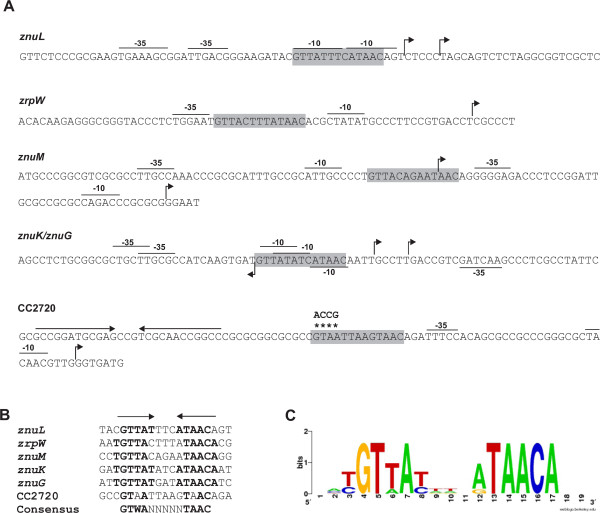
**Localization of Zur binding sequences in the regulatory regions of Zur-regulated genes. (A)** The putative Zur-binding sites predicted *in silico* are shaded. Arrows indicate transcriptional start sites experimentally identified in [39] which allowed the prediction of −35 and −10 promoter elements. Asterisks indicate base changes through site-directed mutagenesis in the putative Zur-binding site at the promoter region of the CC2720-26 operon. **(B)** Alignment of nucleotide sequences containing the putative Zur binding site in the promoter of Zur-regulated genes. Bold letters and arrows indicate inverted repeats sequences used in *in silico* screening for putative Zur-regulated genes. **(C)** DNA sequence logo representing the *C. crescentus* Zur-binding site was obtained by using the WebLogo generator.

The same fragment containing the CC2720-26 promoter was used in an EMSA with *C. crescentus* purified His-FLAG-Zur protein. However, it was not possible to see *in vitro* Zur binding to the DNA probe containing this promoter. In an attempt to determine whether the predicted Zur-binding site of the CC2720-26 promoter was functional and if Zur regulates this operon directly, site-directed mutagenesis was performed. The first half of the conserved motif of the predicted Zur-binding site was changed from GTAA to ACCG, leaving unchanged the remaining of the binding sequence (Figure 4A). This DNA fragment was cloned upstream to the *lacZ* reporter gene and introduced into NA1000 and MM69 strains. This change in sequence led to a decrease in the expression in NA1000 to levels similar to those observed with the wild-type promoter in MM69, making it also unresponsive to zinc (Figure 3B). These results confirmed that the predicted Zur binding site in the regulatory region of CC2720-26 operon is necessary for its regulation in response to zinc ions, likely by direct Zur binding. This was further confirmed by complementation with the *zur* gene *in trans*, which restored the expression pattern observed in the wild type (Figure 3C).

By analyzing the regions upstream of the initiation codons of the validated genes and taking into account the transcription start sites determined by [30], it was possible to predict the positions of the −10 and −35 promoter sequences of these genes. Several of these genes were described as having two promoters, one responding to the cell cycle and the second one to metal stress [30], agreeing with the zinc-dependent regulation observed in this work. The positions of the Zur-binding sites in these regulatory regions are shown in Figure 4A. In *znuK*, *znuL* and *znuGHI* the Zur operator overlaps the −10 region, while in *zrpW* and *znuM* it is located between the −10 and −35 elements. These locations of the Zur operators agree with Zur acting as a repressor of the expression of these genes, confirming the data obtained with the microarray and β-galactosidase assays. Oppositely, in the CC2720-26 promoter region, the putative Zur binding sequence is upstream of the −35 promoter element, agreeing with Zur acting as an activator, data also supported by the previous assays. Alignment of the sequences present in the promoter regions of *znuL*, *zrpW*, *znuM*, *znuK*/*znuGHI* genes and CC2720-26 operon revealed that the *C. crescentus* Zur-binding site is an inverted repeated sequence with two conserved blocks (Figure 4B and C).

### Importance of the Zur-regulated TonB-dependent receptors ZnuK and ZnuL for zinc scavenging

**Figure 5:**
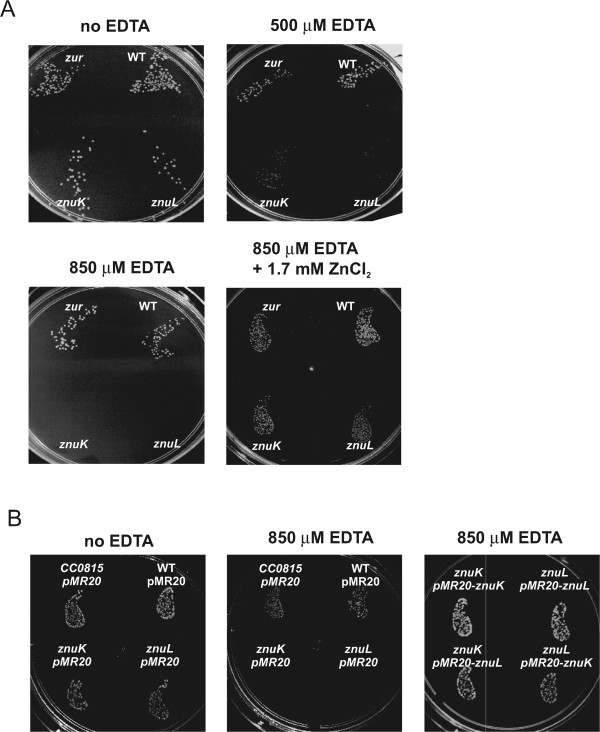
**Contribution of** ***znuK*** **and** ***znuL*** **genes for growth in zinc deprivation. (A)** Growth of NA1000, *zur*, *znuK* and *znuL* mutant strains under different regimens of zinc availability. Aliquots of 20 μL from a 10^−5^ dilution of cultures were plated on M2 medium without or with addition of 500 μM EDTA (3 days growth), 850 μM EDTA (5 days growth) or 850 μM EDTA and 1.7 mM ZnCl_2_ (3 days growth). **(B)** Growth of NA1000, CC0815::mini-Tn5, *znuK*::mini-Tn5 and *znuL*::mini-Tn5 strains harboring plasmid pMR20 containing or not the genes indicated. Aliquots of 20 μL from a 10^−5^ dilution of cultures were plated on M2 medium containing tetracycline (1 μg ml^−1^) and the same additions as in **(A)**.

Among the eight genes upregulated in the *zur* mutant, identified from both microarray analysis and *in silico* screening, three encode putative TonB-dependent receptors (*znuK*, *znuL* and *znuM*). Mutant strains for *znuK*, *znuL* and CC0815 [31] were analyzed, this last one being a gene encoding for a TonB-dependent receptor not responsive to zinc or iron and used as control. All strains were then grown under different conditions of zinc availability, in order to determine the role of ZnuK and ZnuL in zinc uptake (Figure 5).

All mutant strains showed growth rates similar to those of wild-type and *zur* strains in minimal M2 medium (data not shown) and also showed no significant differences in colonies size on M2 agar plates (Figure 5A and B). When EDTA was added to the medium up to a final concentration of 500 μM, *znuK* and *znuL* mutant colonies were smaller than wild-type and *zur* colonies, suggesting slower growth rates in this condition. Additionally, when EDTA concentration was raised up to 850 μM, only wild-type, MM78 (CC0815) and the *zur* strains were able to form colonies after a 5-day incubation, with the *zur* mutant showing larger colonies than NA1000, suggesting that derepression of the zinc uptake systems benefits the *zur* mutant strain under extreme zinc limitation condition. The addition of ZnCl_2_ to the plates containing 850 μM EDTA restored growth of the *znuK* and *znuL* strains, confirming that growth was prevented by the absence of zinc (Figure 5A). Besides that, the ability of MM78 to grow under severe zinc deprivation indicates that the phenotype seen in *znuK* and *znuL* mutant strains is not a general or unspecific result but instead is a specific effect of zinc uptake impairment (Figure 5B). Complementation of each mutation with the respective gene *in trans* restored the ability of growing in 850 μM EDTA (Figure 5B). Interestingly, a cross-complementation with the other gene also allowed growth under these conditions, suggesting that an increased expression of one TonB-dependent zinc transporter may compensate for the lack of the other one.

## Discussion

In this paper, we studied the Zur protein from *C. crescentus*, identified several zinc and Zur-regulated genes and characterized two novel TonB-dependent receptors involved in zinc acquisition. According to the DNA microarray data and *in silico* analysis we defined that the *C. crescentus* Zur regulon is composed by genes mainly involved in uptake and efflux of zinc. The results obtained from EMSA and β-galactosidase assays show that Zur binds directly to the regulatory regions of *znuGHI* (ABC transport system), *znuK*, *znuL*, *znuM* (TonB-dependent receptors) and *zrpW* (putative transporter). Zur acts repressing these genes in the presence of zinc but when this metal is scarce Zur is released from the operators liberating transcription to increase production of zinc uptake systems. Conversely, Zur activates transcription of the CC2720-26 operon in condition of high zinc concentration, allowing expression of the RND and P-type ATPase efflux systems. It was previously demonstrated that the RND system encoded by *czrCBA* is essential for growth in the presence of added zinc or cadmium [29, 31].

Studies performed with many other bacteria have already described that high affinity-ABC transport systems are regulated by Zur and, more recently, studies using transcriptomics analysis [8, 32, 33] and proteomics [11] approaches identified TonB-dependent receptors regulated by Zur in response to zinc, but only the two TonB-dependent receptors from *Neisseria meningitidis* have been characterized as zinc uptake systems [11, 12]. There are few cases described in which Zur acts as a positive regulator of extrusion systems. This ability was first described for Zur from *X. campestris* [08] and later for *C. glutamicum* [09]. Hence, as in those bacteria, Zur is a transcription factor responsible for direct regulation of both zinc uptake and extrusion in *C. crescentus*.

It was not possible to obtain *in vitro* binding of Zur to the promoter of CC2720-26 operon, but the existence of a Zur-binding site on this promoter was confirmed by site-directed mutagenesis. Although all Zur-binding sites in promoters of Zur-repressed genes overlapped the promoter elements as typically observed for transcriptional repressors, the position of the Zur-binding site in CC2720-26 was upstream of the −35 promoter element in agreement with its role as a positive transcriptional regulator. In the regulatory region of CC2720-26 there is an imperfect inverted repeat 11-4-11 (GCCGGATGCGA-4 N-TCGCAACCGGC) located 13 nt upstream of the Zur-binding site. In *X. campestris*, Zur activates a gene encoding a transporter belonging to the CDF family in response to zinc, and an imperfect inverted repeat of 20-bp is found on the promoter region which is essential for binding of Zur both *in vivo* and *in vitro*, indicating that Zur recognizes simultaneously that inverted repeat and the Zur binding sequence [08]. We speculate that the inability of *C. crescentus* Zur to bind the CC2720-26 promoter *in vitro* could be due to the requirement for an accessory protein that binds to the inverted repeat sequence and stabilizes Zur binding.

In addition to the genes directly regulated by Zur, microarray and *in silico* analysis revealed that most of the genes downregulated in MM69 strain with no predicted Zur-binding sites belongs to the Fur regulon, being repressed by iron-bound Fur in the presence of iron [26]. One hypothesis to explain these results is that in the *zur* mutant background the increased intracellular zinc concentration could result in mismetallation of Fur by zinc ions and the zinc-bound Fur protein could be inappropriately repressing Fur-regulated genes in an iron-independent manner. Indeed, it has already been shown that Fur bound to other divalent cations as zinc, cobalt and manganese preserves the ability of binding to DNA *in vitro* with almost the same affinity than the iron-bound Fur [34, 35]. An example that zinc may metallate Fur in its regulatory site is the crystal structure of the Fur protein from *Pseudomonas aeruginosa* where the regulatory site was occupied by a zinc ion instead of an iron ion [36]. Therefore, our results suggest cross-talk between Zur and Fur caused by an unbalance in zinc levels in the *zur* mutant, which corroborates previous findings that metal ion selectivity can be disturbed among metalloregulators of the Fur family [35, 37].

Remarkably, the *C. crescentus* Zur regulon does not contain genes encoding ribosomal proteins. It has been proposed for *B. subtilis* [38, 39], *X. campestris* [08], *Yersinia pestis* [22], *Mycobacterium tuberculosis* [40], *N. meningitidis* [32] and inferred via *in silico* analysis for several other organisms [41] that some ribosomal proteins could act as a zinc reserve. In conditions of zinc deficiency, their expression would be repressed in detriment of its paralogs that did not use zinc in their structure. *C. crescentus* does not have paralogs of genes encoding these ribosomal proteins, indicating that this mechanism is not used. However, despite the fact that in *C. crescentus* ribosomal proteins are not Zur-regulated, we cannot exclude the possibility that they might still act as intracellular zinc reserves under control of another zinc regulator. The zinc and Zur-repressed gene z*rpW*, encoding a putative transporter, is likely cotranscribed with CC0321 (*zrpX)*. The hypothetical protein ZrpX has two conserved CobW domains in the N- and C- terminal portions. This protein belongs to COG0523 family which in some cases was observed to be Zur-regulated. It is believed that proteins belonging to the Zur-regulated subfamily of COG0523 family may perform metallochaperone functions related to intracellular traffic and storage of zinc [42]. Thus, in *C. crescentus* ZrpX could be an alternative to the ribossomal proteins as a zinc reserve.

According to previous and current data it is likely that Zur is not the solely responsible for zinc homeostasis in *C. crescentus.* The putative zinc transporter CztA and its regulator CztR previously identified [28] are zinc responsive and not regulated by Zur. Moreover, the *nczCBA* RND system involved in zinc extrusion is upregulated in response to zinc [29] also in a Zur-independent manner. Genome analysis predicts the existence of other transcriptional regulators from the ArsR/SmtB and MerR families, already described as involved in the regulation of genes for metal extrusion in other organisms [06, 07, 25]. Further studies with global approaches might elucidate the role of these regulators in zinc homeostasis.

As Zur represses the expression of zinc uptake systems and activates the expression of two efflux systems, there should be an accumulation of intracellular zinc in the *zur* mutant. Indeed, strain MM69 showed 10% more intracellular zinc content than wild-type (normalized to dry mass), which may be a result of constitutive uptake and low efflux in the absence of Zur. However, it must be taken into account that *C. crescentus* NA1000 encodes in its genome seven RND systems, two CDF proteins and three P-type ATPases [25, 43] which also may have some role in zinc efflux. A second RND system, *nczCBA* - which is not Zur-regulated - is induced in zinc excess and the *nczA* mutant showed impaired growth in the presence of 130 μM ZnCl_2_ [29]. Therefore, it is possible that the *zur* mutant strain did not show a more pronounced accumulation of intracellular zinc because other efflux systems as *nczCBA* may be eliminating zinc.

Many nutrients and ions can cross the bacterial outer membrane and enter into the periplasmic space through passive transport via porins. However, when the concentration of an ion is very low outside, diffusion becomes unfavorable. Thus, TonB-dependent receptors can play a crucial role in the uptake of scarce metals. Although the role of these proteins in iron-complex uptake is well-defined, little is known about others metals [44], with only few reports describing the role of TonB-dependent receptors in the uptake of nickel and zinc [11, 12, 45]. In this work, we presented experimental evidence that two out of the three Zur-regulated TonB-dependent receptors, ZnuK and ZnuL, are important for *C. crescentus* growth under zinc deprivation. Moreover, cross complementation analysis showed that there seems to be a functional redundancy between *znuK* and *znuL*, which will be further investigated*.* Since *C. crescentus* is well-adapted to poor nutrient conditions, and has three receptors of this type as opposed to only one in pathogenic *N. meningitidis,* the uptake of zinc would be less strongly impaired by the absence of a single transporter.

## Conclusions

This work has characterized the regulon of Zinc Uptake Regulator (Zur) of *Caulobacter crescentus* by means of transcriptomics and *in silico* analyses. The Zur regulon comprises genes encoding transport proteins, such as ABC transporters, TonB-dependent receptors, an RND system and other putative transporters. Our results showed that Zur can act both as a repressor of genes for zinc uptake (*znuK*, *znuL*, *znuM*, *znuGHI* and *zrpW)*, and as an activator positively regulating the efflux systems encoded by *czrCBA* and *zntA*. Importantly, the highly expressed TonB dependent transporters ZnuK and ZnuL were shown to be essential for growth under zinc starvation, acting as key zinc scavenging systems. It was observed that Zur plays a significant role in keeping zinc homeostasis, since its absence lead to an alteration in the expression of some genes belonging to the Fur regulon. This is probably due to the unbalance in zinc intracellular concentration, which may cause further effects in other metal responsive regulatory systems. Further studies will be carried out in order to establish the whole regulatory network in response to metal availability in *C. crescentus.*

## Methods

### Bacterial strains and growth conditions

*C. crescentus* NA1000 wild-type strain and its derivative strains MM69 (Δ*zur*), MM72 (*znuK*::mini-Tn5 Km2), MM73 (*znuL*::mini-Tn5 Km2) and MM78 (CC0815::mini-Tn5 Km2) were grown at 30°C under agitation in minimal medium M2 [46] and, when necessary, supplemented with tetracycline (1 μg ml^−1^), kanamycin (5 μg ml^−1^) or nalidixic acid (20 μg ml^−1^). MM72, MM73 and MM78 strains were obtained from a mutant library generated by mini-Tn5Km2 transposon insertion [31] and in the present study these mutations were individually transduced into a fresh *C. crescentus* NA1000 background. Complementation of the mutations was obtained by introduction of the low copy plasmid pMR20 [47] carrying either *znuK* or *znuL*. Zinc depletion and abundance were obtained by supplementing media with ethylenediamine tetraacetic acid (EDTA) or ZnCl_2_, respectively, as specified in each experiment. *E. coli* strains were grown at 37°C under agitation in Luria-Bertani (LB) or M9 media and, when necessary, supplemented with tetracycline (12.5 μg ml^−1^) or kanamycin (50 μg ml^−1^). All plasmids were introduced into *Caulobacter* strains via conjugation with *E. coli* strain S17-1 [48].

### Construction of *zur*mutant strain and complementation

A *zur* mutant strain was obtained by allelic replacement in strain NA1000. Two 920-bp DNA fragments upstream and downstream from CC0357 [GenBank: AAK22344] respectively were cloned *in tandem* into the suicide vector pNPTS138 (a kind gift of D. Alley), and the construction was used for deletion of *zur*, generating strain MM69. Gene deletion was confirmed by both PCR and Southern blotting (not shown). Complementation of the *zur* mutation was obtained by introduction of plasmid pUJ142 [49] carrying an 1110-pb DNA fragment containing the *zur* gene.

### RNA extraction and DNA microarray analysis

RNA purification from *C. crescentus* NA1000 and MM69 strains was carried out from 50 ml midlog phase cultures grown in M2 medium supplemented with 200 μM ZnCl_2_ for 1 h. Total RNA was extracted using Trizol Reagent (Invitrogen), according to the manufacturer’s instructions and residual chromosomal DNA contamination was removed by RNase-free DNase I (Fermentas) treatment followed by precipitation using sodium acetate and ethanol. Purified RNA underwent spectrophotometric quantification and visualization on formaldehyde agarose gels. RNA samples isolated from three independent bacterial cultures for each strain were used for DNA microarray analysis.

The DNA microarray experiments were performed as described in [27], using a custom-designed DNA oligo array (Agilent Technologies). The arrays contain 9–11 probes covering the region around the start codon (−300 to +200) of each predicted ORF in the genome of *C. crescentus* CB15. The four last probes for each gene, which correspond to the beginning of the translated region, were the ones considered for the expression analysis. Briefly, cDNA was generated from 12.5 μg of total RNA and labeled with either Cy3 or Cy5 fluorescent dyes using the FairPlay III Microarray Labeling System (Stratagene). Labeled cDNA samples were hybridized to the probes, the arrays were scanned with an Agilent High Resolution Microarray Scanner and the data were extracted and normalized with the Feature Extraction Software 9.0 (Agilent). The values for the relative expression of each gene were the average of the values of its four last probes in all three biological replicates. We included as differentially expressed genes (Table 1) those that meet the following criteria: a minimum of 2-fold expression change for at least three probes in at least two biological replicates. The microarray data have been deposited in the Gene Expression Omnibus (GEO) database (http://www.ncbi.nlm.nih.gov/geo/) under accession number GSE57136.

### β-Galactosidase activity assays

Zur and zinc-dependent gene regulation were determined using transcriptional fusions of promoters with the *lacZ* reporter gene in pRK*lacZ*290 plasmid [50]. Regulatory regions of the CC0214, CC0320, CC1517 genes were amplified by PCR using primers 0214–1 (5′-GGAATTCTTCGCGCGCCGAAAGTGAC-3′) and 0214–2 (5′-CGGATCCAACCACGAGATGCGCGGTC-3′), Pcc0320F (5′-TATGAATTCTCGAGCTTTTCGACCACCAGC-3′) and Pcc0320R (5′-TATGGATCCCGACCAGCACGAACGGCAGC-3′), 1517–1 (5′-GGAATTCGCCTTGTTGATCTCGTCAG-3′) and 1517–2 (5′-GGATCCGTCTGGATCTCACTGGAGAA-3′), respectively. These PCR products were cloned into pGEM-T Easy, sequenced and subcloned as *EcoR*I/*BamH*I fragments into pRK*lacZ*290. The promoter region of CC0663 was obtained by subcloning a 512-bp *EcoR*I/*BamH*I fragment from pNPTS138Δ*cspB* [51] into pRK*lacZ*290. The CC1518 promoter fusion was obtained by inverting the fragment cloned into pRK*lacZ*290 from CC1517. The CC2720 promoter fusion was already available [29]. These fusions were introduced into NA1000 and MM69 strains by conjugation. Cultures were grown in M2 medium up to an optical density of 0.4, supplemented with either 500 μM of ZnCl_2_, 500 μM of EDTA or 500 μM of both, and then incubated for 2 hours. Gene expression was measured by determining β-galactosidase activity as described by [52].

### *In silico*analysis of Zur binding sites

Four Zur-binding motifs described for different proteobacteria [08, 22, 32, 33] were aligned in order to identify conserved sequences, which were in turn used to perform a genome-scale screening for putative Zur-binding sites in the *C. crescentus* genome sequence in the RSAT website [53]. Additionally, promoter regions of Zur-regulated genes, identified by microarray analysis, were searched for conserved DNA motifs, using the MEME tool [54]. Sequence logos were generated using WebLogo [55].

### Site-directed mutagenesis

Site-directed mutagenesis of the CC2720 promoter region was performed using primers CC2720-F (5′-GCGATTGGCTAACG-3′) and CC2720-Zur-boxmut-R (5′-AATCTGTTACTTCGCGGTTGCGCGCCGCGCG-3′), CC2720-Zur-boxmut-F (5′-CGCGCGGCGCGCAACCGCGAAGTAACAGATT-3′) and CC2720-R (5′-GACCAACGCAACCAAG-3′) as described previously for Fur-box mutagenesis [26]. The resulting 500-bp PCR products were cloned into pGEM-T Easy, sequenced to confirm substitution of GTAA by ACCG nucleotides in the Zur-binding site, and subcloned as *EcoR*I/*BamH*I fragment into pRK*lacZ*290. This construction was then used for β-galactosidase activity assays.

### Zur protein expression and purification

The *C. crescentus zur* coding region was amplified from genomic DNA by PCR, using primers Zur-expresstag1 encoding a 5′-FLAG sequence (5′- TTCATATGGATTACAAGGATGACGATGACAAGATGAGCATGGCCAACGCTCCC-3′)/Zur-express2 (5′-TTGGATCCTCAGCTTCGGCAATCCGCGC-3′), and the 419-bp *Nde*I/*BamH*I fragment was cloned into pET28a plasmid (Novagen). This construction was introduced into *E. coli* BL21(DE3) strain and His-FLAG-Zur protein expression was induced for 3 h with 0.5-mM IPTG added to midlog cultures grown at 30°C in M9 medium. The recombinant protein was purified from soluble extract by NTA-resin affinity chromatography (Qiagen).

### Electrophoretic mobility shift assay (EMSA)

PCR fragments containing the promoters of selected genes CC0214, CC0320, CC0663, CC1517/CC1518 were purified and labeled with [γ-32P]-ATP using T4 Polynucleotide Kinase (Fermentas). Unincorporated nucleotides were removed with Qiaquick PCR purification kit (Qiagen). DNA binding was performed in a 20 μl reaction in binding buffer (10 mM Tris–HCl pH 7.5, 40 mM KCl, 1 mM MgCl_2_, 1 mM dithiothreitol, 0.1 mM MnCl_2_, 0.1 mg ml^−1^ bovine serum albumin, 5% glycerol), containing salmon sperm DNA (0.1 mg ml^−1^), labeled DNA probes and increasing amounts of purified His-FLAG-Zur protein (0, 25, 50, 100, 200 and 500 nM). In competition assays, a 30-fold excess of cold probe was used to challenge each of the labeled probes. The probe containing the coding region of the stationary phase-induced gene *cspD* was used as the non-specific competitor. After incubation at 30°C for 30 min, the samples were loaded onto a native 4%, 5% or 8% polyacrylamide gels (depending on probe size) in 40 mM Tris-acetate buffer pH 8.0 (with no added EDTA), containing 2 mM MnCl_2_. Gels were dried and radioactive species were detected by autoradiography.

### Measurement of zinc content in cell

Overnight cultures were diluted to OD 0.1 in 500 ml M2 medium and incubated at 30°C under agitation. When cultures reached OD 0.4, 200 μM ZnCl_2_ was added and cultures were incubated for further 2 h. Cells from cultures were harvested and washed twice with wash buffer (0.5 g l^−1^ Na_2_PO_4_, 1.74 g l^−1^ KH_2_PO_4_ and 1.06 g l^−1^ NH_4_Cl pH 6.9) containing 500 μM EDDS (Sigma) to remove externally bound or free remaining zinc. After washes, cultures were harvested, dried and then metal content was determined by Inductively Coupled Plasma Optical Emission Spectrometry (ICP-OES) method, normalized to dry mass.

### Phenotypic characterization of *znuK*and *znuL*mutants under zinc starvation

To determine the effect of zinc starvation on viability of NA1000, MM69, MM72, MM73 and MM78 strains, they were grown in M2 medium at 30°C up to midlog phase (OD600 nm 0.6) and aliquots were taken for serial dilutions. 20 μl-aliquots of a 10^−5^ dilution were spread on four M2 agar plates, containing either 500 μM, 850 μM EDTA, 850 μM EDTA plus 1.7 mM ZnCl_2_ or no additions. Plates were incubated for 72 h or 120 h and then photographed.

## Electronic supplementary material

Additional file 1: Figure S1: Amino acid alignment of *C. crescentus* Zur with other regulators of the Fur family. (PDF 195 KB)
