# Supplementary material for: Analysis of the Caulobacter crescentus Zur regulon reveals novel insights in zinc acquisition by TonB-dependent outer membrane proteins
Source: BMC Genomics. 2014 Aug 28;15(1):734. doi: 10.1186/1471-2164-15-734 (PMC4176598; doi:10.1186/1471-2164-15-734)
Supplement: Supplementary file 1 — Additional file 1: Figure S1: Amino acid alignment of C. crescentus Zur with other regulators of the Fur family. (PDF 195 KB) [file 12864_2014_6470_MOESM1_ESM.pdf]

```
                       *        20          *         40
EC_ZUR : --------------------MEKTTTQELLA----QAEKICAQRN :  21
YP_ZUR : --------------------MNPINQEKLLA----QAESLCQQRN :  21
XC_ZUR : MTKHTHTQTHDQEHACTAPHHHVDDANSFVR----AVERACSERG :  41
PA_ZUR : -----MYKIAPKTPLACQPHDHSQCVSTALA----EADALCARQG :  36
CC_ZUR : -------MSMANAPSCGHDHHNHGVAGAALAAELDAAEARCAAAD :  38
CC_FUR : ----------------------------MDR------LEKACIEKG :  12
SC_NUR : --------------------MVSTDWKSDIR-----------QRG :  14

                  *        60         *         80          *
EC_ZUR : VRLTPQRLEVLRLMSLQDGAISAYDLLDLLREAEPQ--AKPPTVY :  64
YP_ZUR : VRLTPQRLEVLRLMAQQPGAISAYDLLDLLRVAEPQ--AKPPTVY :  64
XC_ZUR : LRLTPIRANVLRLIADAGKPVKAYELLDWVREGKGVGADAPPTVY :  86
PA_ZUR : VRLTELRRRVLELVWQSHKPLGAYDILAVLSETDGR-RAAPPTVY :  80
CC_ZUR : QRLTAPRRRVLELLLEAGQPVKAYDLISSFGGSGPP--AKPPTVY :  81
CC_FUR : MRMTDQRRVIARVLSSAEDHPDVEELHRRAHAIDPH--ISIATVY :  55
SC_NUR : YRLTPQRQLVLEAVDT-LEHATPDDILGEVRKTASG--INISTVY :  56

                 100         *        120         *
EC_ZUR : RALDFLLEQGFVHKVESTNSYVLCHLFDQPTHTSAMFICDRCGAV : 109
YP_ZUR : RALDFLLEQGFIHRVESANSYVLCHHFEEPTHTSALFICDRCKIV : 109
XC_ZUR : RALDFLMANGFVHKLESVNAFVACHHPNSAQHSVPFLICDRCHSA : 131
PA_ZUR : RALDFLQENGLVHRIASLNAFVGCNNP-EHSHQGQFLICRTCHTA : 124
CC_ZUR : RALDFLEKQGFAHRIESLNAYVACRKE-ADGHAAAFLICDCCGAT : 125
CC_FUR : RTVRLFEESGIIERHDFRDGRS--RYEQSPDHHHDHLIDMKTGKV :  98
SC_NUR : RTLELLEELGLVSHAHLG--HGAPTYHLADRHHHIHLVCRDCTNV :  99

             140         *        160         *        180
EC_ZUR : KEECAEGVEDIMHTLAAKMGFALRHNVIEAHGLCAACVEVEACRH : 154
YP_ZUR : TERPTVGIEEALAQLAKQSGFTLRHSVVEAHGLCTECGVVEACES : 154
XC_ZUR : VELEDRDVVSQLEARAKALGFQPQAQTLEVHGLCAKCAAAG---- : 172
PA_ZUR : IELEQPDISRAIVAGANSVGFAVESQTVEVVGLCGTCRDQKDA-- : 167
CC_ZUR : REIEP-KASAEIIAAGEAAGYALTGVTIEAHGLCADCRS------ : 163
CC_FUR : VEFVDEEIEALQHAIAKKLGYKLIDHRLELYGMPLEE-------- : 135
SC_NUR : IEADLSVAADFTAKLREQFGFDTDMKHFAIFGRCESCSLKGSTTD : 144

                       *
EC_ZUR : PEQCQHDHSVQVKKKPR : 171
YP_ZUR : HDDCEHDHSIVVKKK-- : 169
XC_ZUR : ----------------- :   -
PA_ZUR : ----------------- :   -
CC_ZUR : ----------------- :   -
CC_FUR : ----------------- :   -
SC_NUR : S---------------- : 145
```

Figure S1 – Amino acid alignment of *C. crescentus* Zur with other regulators of the Fur family. Uptake regulators for zinc (ZUR), iron (FUR) and nickel (NUR) are shown. The GenBank no. and abbreviations are as follows: EC, *Escherichia coli* [AAC77016]; YP, *Yersinia pestis* [AAS60741]; XC, *Xanthomonas campestris* [AAY48498]: PA, *Pseudomonas aeruginosa* [AAG08884]; CC, *Caulobacter crescentus* [Zur, AAK22344; Fur, AAK22044]; SC, *Streptomyces coelicolor* [CAB94084].
